# CpG oligodeoxynucleotide reduces PrP^Sc^ accumulation and prolongs survival in prion-infected mice

**DOI:** 10.1016/j.mocell.2026.100335

**Published:** 2026-03-03

**Authors:** Mohd Najib Mostafa, Byungki Jang, Mo-Jong Kim, Myung-Ju Choi, Younghee Lee, Yong-Sun Kim, Hyung-Joo Kwon, Eun-Kyoung Choi

**Affiliations:** 1 Department of Biomedical Gerontology, Graduate School of Hallym University, Chuncheon, Gangwon-do 24252, Republic of Korea; 2 Ilsong Institute of Life Science, Hallym University, Yeongdeungpo-gu, Seoul 07247, Republic of Korea; 3 Department of Biochemistry, College of Natural Sciences, Chungbuk National University, Cheongju, Chungbuk 28644, Republic of Korea; 4 Department of Microbiology, College of Medicine, Hallym University, Chuncheon, Gangwon-do 24252, Republic of Korea; 5 Institute of Medical Science, College of Medicine, Hallym University, Chuncheon, Gangwon-do 24252, Republic of Korea

**Keywords:** Autophagy, AMP-activated protein kinases, Cytosine-phosphate-guanine oligonucleotides, Neurodegeneration, Prion clearance, Prion disease

## Abstract

Prion diseases, also known as transmissible spongiform encephalopathies, are characterized by the accumulation of misfolded prion proteins (PrP^Sc^), which resists clearance due to impaired removal mechanisms and immune tolerance. Although strategies aimed at enhancing intracellular clearance pathways, including autophagy, have shown promise, effective in vivo interventions remain limited. Innate immune stimulation with CpG oligodeoxynucleotides (CpG ODN), a Toll-like receptor 9 agonist that mimics bacterial DNA containing unmethylated CpG motifs, has been reported to extend survival in prion-infected mice; however, the underlying mechanisms involved remain incompletely defined. In this study, we examined the effects of CpG ODN in both in vitro and in vivo models of prion disease using the 22L scrapie strain. Mice treated with CpG ODN prior to infection exhibited prolonged mean survival compared with vehicle-treated controls and showed reduced PrP^Sc^ accumulation in spleen and brain tissues at 60 and 170 days post-infection, respectively. These changes were correlated with increased AMPK (Tyr172) phosphorylation and alterations in autophagy-associated signaling markers in the brain tissue. In 22L scrapie-infected neuronal cells, CpG ODN treatment significantly reduced PrP^Sc^ levels, and this effect was attenuated by pharmacological inhibition of autophagy-associated and lysosomal degradative processes. Collectively, these findings indicate that CpG ODN reduces PrP^Sc^ accumulation and prolongs survival in prion-infected mice, supporting the involvement of autophagy-associated degradative processes without establishing enhanced autophagic flux.

## Graphical Abstract

**Figure:**
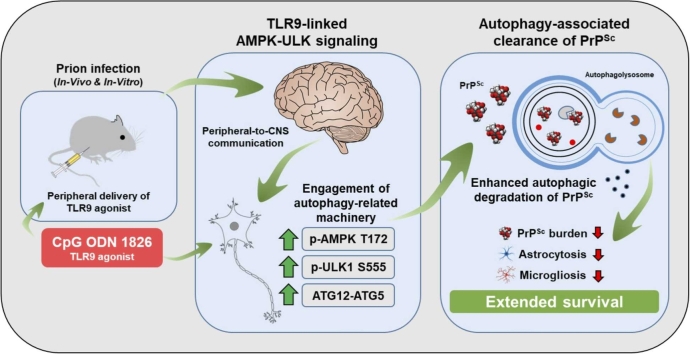

## BACKGROUND

Prion diseases, or transmissible spongiform encephalopathies, are a group of rare, fatal neurodegenerative disease that affect both humans and animals. These diseases are characterized by the accumulation of an abnormally folded isoform of the cellular prion protein (PrP^C^), termed PrP^Sc^. This pathological form is enriched in β-sheet structures, making it prone to aggregation and resistant to proteolytic degradation. The conversion of PrP^C^ to PrP^Sc^ is central to disease pathogenesis, yet the exact molecular triggers of this conformational change remain poorly understood. PrP^C^ is a glycoprotein predominantly expressed in the central nervous system (CNS), although it is also found in peripheral tissues (Prusiner, 1998). Under physiological conditions, PrP^C^ plays roles in synaptic transmission, neuroprotection, circadian rhythm regulation, and cellular signaling (Castle and Gill, 2017). Upon conversion to PrP^Sc^, the protein adopts an insoluble, aggregation-prone structure that initiates a cascade of neurotoxic events, including neuronal cell death, astrocyte and microglial activation, and spongiform changes in brain tissue, ultimately leading to severe cognitive and motor dysfunction (Outeiro and Vieira, 2025).

Prion diseases can occur through inherited mutations in the PRNP gene, spontaneous protein misfolding events, or acquired infections via contaminated food, surgical instruments, or biological products. In acquired prion infections, such as those induced experimentally in animal models, the initial accumulation of PrP^Sc^ occurs in peripheral lymphoid tissues—including the spleen and lymph nodes—before neuroinvasion. The infectious agent then travels via peripheral nerves to the spinal cord and ultimately the brain (Aguzzi, 2003, Béringue et al., 2020, DeFranco and Telling, 2025).

An unusual and significant aspect of prion pathogenesis is its interaction with the host immune system. Despite being a proteinopathy, prion diseases do not typically elicit a strong adaptive immune response, as PrP^Sc^ is a conformational variant of a self-protein. Nevertheless, components of the immune system, particularly the follicular dendritic cells, play crucial roles in early prion replication. PrP^Sc^ accumulates in germinal centers on follicular dendritic cells, a process dependent on the expression of PrP^C^ by these stromal cells (Brown et al., 1999, McCulloch et al., 2011). Moreover, innate immune cells such as macrophages and dendritic cells can internalize prions, although their ability to clear or degrade PrP^Sc^ is limited due to the protease-resistant nature of the aggregates (Beringue et al., 2000, Carp and Callahan, 1981, Luhr et al., 2002). Several cellular clearance mechanisms, including the unfolded protein response, proteasomal degradation, and autophagy, have been implicated in attempts to manage PrP^Sc^ accumulation. Nevertheless, these mechanisms ultimately fail to prevent rapid accumulation of PrP^Sc^, leading to neurodegeneration and the onset of prion-associated symptoms (Goold et al., 2015).

Modulating the innate immune response has emerged as a potential therapeutic strategy to combat prion diseases. Toll-like receptors (TLRs), especially TLR9, play important roles in the recognition of microbial components and the initiation of immune responses. Synthetic cytosine-phosphate-guanine oligodeoxynucleotides (CpG ODNs), which mimic bacterial DNA motifs and activate TLR9, have been used to stimulate innate immunity in various disease contexts, including cancer, infections, and neurodegenerative disorders (Deng et al., 2004, Shirota et al., 2015). In prion disease models, CpG ODN treatment has been associated with delayed onset of symptoms and prolonged survival (Sethi et al., 2002). However, the underlying mechanisms through which CpG ODNs exert their protective effects remain incompletely understood.

Apart from immunomodulatory effects, emerging evidence suggests that TLR9 stimulation can influence autophagic pathways in non-immune cells, particularly through the activation of AMP-activated protein kinase (AMPK), a key regulator of cellular energy homeostasis and autophagy induction (Shintani et al., 2013, Ye et al., 2018). AMPK activation inhibits the mechanistic target of rapamycin (mTOR), thereby promoting autophagy. Through this pathway, CpG ODNs, as TLR9 agonists, may facilitate the clearance of PrP^Sc^ and other toxic aggregates, potentially attenuating disease progression.

In this study, we investigated the immunomodulatory and neuroprotective potential of CpG ODNs in a murine model of prion disease. Using BALB/c mice infected with the 22L prion strain, we assessed survival outcomes following a single intraperitoneal injection of CpG ODNs. To elucidate the underlying mechanisms, we examined key molecular markers associated with autophagy and AMPK signaling in both brain tissue and a scrapie-infected neuronal cell model. Our findings demonstrate that CpG ODN treatment prolongs survival, attenuates neuropathological changes, and reduces PrP^Sc^ accumulation in association with activation of AMPK signaling and engagement of autophagy-related pathways.

## MATERIALS AND METHODS

### Animals

Five-week-old female BALB/c mice were originally purchased from RAON Bio and housed under specific pathogen-free conditions at the animal facility of the Ilsong Institute of Life Science, Doheon Research Center (Seoul, Korea), for the development of prion disease animal models. All procedures for the animal experiments were performed according to the Guide for the Care and Use of Laboratory Animals of the National Veterinary Research and Quarantine Service of Korea, with the approval of the Institutional Animal Care and Use Committee of Hallym University (HMC 2022-0-1014-42, HMC 2023-0-0914-32).

### CpG ODN Treatment and Scrapie Infection

CpG ODN 1826 (type B, TLR9 ligand) and TLR9 antagonist (ODN 2088) were purchased from InvivoGen (San Diago, CA, USA) containing phosphorothioate-modified backbones. The sequences used were as follows: CpG ODN 1826, 5′-TCCATGACGTTCCTGACGTT-3′ and ODN 2088, 5′-TCCTGGCGGGGAAGT-3′. ODNs were dissolved in distilled water (DW) at a concentration of 1 mg/ml. BALB/c mice were intraperitoneally (i.p.) injected with either 50 μg of CpG ODN 1826 or an equal volume of DW as a control (Kim et al., 2018). For scrapie infection, mice were i.p. inoculated 7 days later with 50 µl of 1% (w/v) brain homogenate in phosphate-buffered saline (PBS, pH 7.4) prepared from 22L scrapie-infected mice. Control mice received normal (non-infected) brain homogenate (NBH) prepared using the same procedure. The incubation period was defined as the interval from the day of inoculation to the onset of terminal clinical signs. For survival curve analysis, mice were divided into 4 groups, (1) Control (DW/NBH), (2) 22L-infected (DW/22L), (3) one-time CpG pre-exposed 22L infected (CpG/22L), and (4) CpG pre-exposed 22L infected mice with 4 additional weekly booster doses post-infection (CpG/22L/CpG). Mice were monitored daily, and survival was assessed using the Kaplan-Meier method in GraphPad Prism 8. Statistical comparisons between groups were performed using the log-rank (Mantel-Cox) test, with significance set at *P* < .05. For molecular and histological analyses, 3 groups were selected: (1) DW/NBH, (2) DW/22L, and the (3) CpG /22L. Mice were sacrificed at 60 dpi and 170 dpi to represent early and preclinical stages. Brain and spleen tissues were collected for PrP^Sc^ detection, molecular marker analysis, and histopathological evaluation.

### Mouse Hippocampal Neuronal Cell Culture and Maintenance of Scrapie-Infected Cells

The mouse hippocampal neuronal cell line ZW13-2 (expressing wild-type PrP), previously established (Kim et al., 2005), was cultured in Dulbecco’s modified Eagle’s medium (DMEM; Biowest, Nuaillé, France) supplemented with 10% heat-inactivated fetal bovine serum (FBS; Biowest), 100 units/ml penicillin, and 100 µg/ml streptomycin (Thermo Fisher Scientific, Rockford, IL, USA), maintained at 37 ℃ in a humidified 5% CO_2_ Incubator. ZW13-2 cells were persistently infected with either 0.01% 22L scrapie-infected brain homogenate (ZW-22L) or normal brain homogenate (ZW-NBH), as previously described (Kim et al., 2020). Infected cells were maintained in Opti-MEM (Sigma-Aldrich, St Louis, MO, USA) supplemented with 10% FBS and subcultured every 3 days at a 1:2 ratio for the first 10 passages. The infected cells stably produced PrP^Sc^ for over 50 passages. Cells were then treated with CpG ODN at concentrations of 1 or 3 μM for 3 or 6 hours in the absence or presence of the autophagy inhibitor bafilomycin A1 (Sigma-Aldrich) or wortmannin (Sigma-Aldrich). For ODN 2088 treatment, cells were treated with 5 μM concentration 1 hour prior to CpG ODN treatment.

### Western Blot Analysis

Spleen and brain tissues from control and 22L scrapie-infected mice, with or without CpG ODN treatment, were homogenized in modified RIPA buffer (50 mM Tris-HCl, pH 7.5, 150 mM NaCl, 1 mM EDTA, 1% Nonidet P-40, 0.5% sodium deoxycholic acid, and 0.1% sodium dodecyl sulfate), supplemented with a protease inhibitor cocktail (Pierce Biotechnology, Rockford, IL, USA), 1 mM Na_3_VO_4_, and 1 mM NaF (Pierce Biotechnology). Cells were washed with ice-cold PBS and lysed in the same buffer. Tissue and cell lysates were centrifuged at 15,000 g for 15 minutes at 4 °C, and protein concentrations were determined using a BCA assay kit (Pierce Biotechnology). Equal amounts of protein (40 μg/lane) were subjected to 12 or 15% SDS-PAGE gels and transferred onto polyvinylidene difluoride membranes (Millipore, Billerica, MA, USA) using an electrotransfer system (Bio-Rad Laboratories, Hercules, CA, USA). Membranes were blocked with 5% nonfat dry milk in PBST (8 mM Na_2_HPO_4_, 2 mM KH_2_PO4, 138 mM NaCl, 2.7 mM KCl, 0.1% Tween 20; pH 7.4) for 1 hour at room temperature (RT), followed by overnight incubation at 4 °C with the following primary antibodies: mouse monoclonal anti-PrP 3F10 (1:2000) (Choi et al., 2006), rabbit polyclonal anti-TLR9 (1:2000, Abcam, Cambridge, UK), rabbit polyclonal anti-phospho AMPK T172 (p-AMPK T172) (1:2000, Cell Signaling Technology, Danvers, MA, USA), rabbit monoclonal anti-AMPK (1:2000, Cell Signaling Technology), rabbit polyclonal anti-phospho-p62 S403 (p-p62 S403) (1:2000, Cell Signaling Technology), rabbit monoclonal anti-p62 (1:2000, MBL, Nagoya, Japan), rabbit monoclonal anti-phospho-ULK1 S555 (p-ULK1 S555)(1:2000, Cell Signaling Technology), rabbit polyclonal anti-ULK1 (1:2000, Cell Signaling Technology), rabbit polyclonal anti-LC3 I/II (1:2000, Cell Signaling Technology), rabbit polyclonal anti-GFAP (1:2000, CosmoBio, Tokyo, Japan), rabbit polyclonal anti-ATG12 (1:2000, Cell Signaling Technology) and mouse monoclonal anti-β-actin (1:2000, Sigma-Aldrich). Membranes were washed 3 times with PBST (10 minutes each) and then incubated for 1 hour at RT with the following horseradish peroxidase-conjugated secondary antibodies: goat anti-mouse IgG or goat anti-rabbit IgG (both 1:5000, Enzo Life Sciences, Farmingdale, NY, USA). For the detection of PrP^Sc^, samples were digested with proteinase-K (PK) at 20 μg/ml for brain tissue or 2 μg/ml for cell lysates for 1 hour at 37 °C prior to incubation with the mouse monoclonal anti-PrP antibody 3F10 (1:2000). Immunoreactive bands were visualized using a chemiluminescent substrate (ATTO, Tokyo, Japan) and imaged with an ImageQuant LAS 4000 apparatus (GE Healthcare Life Sciences, Piscataway, NJ, USA). Band intensities were quantified using Image J software (NIH), and statistical analyses were performed using GraphPad Prism 8 (GraphPad Software, San Diego, CA, USA).

### Hematoxylin and Eosin (H&E) Staining

Tissue sections were prepared for histopathologic examination as previously described (Kim et al., 2024). Briefly, brain tissues were collected at 170 dpi from control (DW/NBH) and 22L-infected mice with vehicle (DW/22L) or CpG ODN (CpG/22L) mice, fixed in 10% neutral-buffered formalin followed by paraffin embedding, and sectioned intro 6 µm-thick sections. The slides were dried overnight, deparaffinized in xylene, and rehydrated through a graded ethanol series. The sections were then stained with ClearView Hematoxylin (BBC biochemical #MA010181, Eleanor Lane, Mount Vernon, USA) and counterstained with Eosin Y solution (BBC biochemical #3610MIRA01, Eleanor Lane, Mount Vernon, USA). After staining, the sections were dehydrated through graded ethanol, cleared in xylene, and mounted using mounting medium. Stained sections were examined under a light microscope (Olympus BX51, Tokyo, Japan).

### Immunohistochemistry and Immunofluorescence

For immunohistochemical staining, neutral-buffered formalin-fixed mouse brains were sectioned at 6 µm-thickness, deparaffinized in xylene, hydrated in a graded ethanol series, and subjected to antigen retrieval in 10 mM sodium citrate buffer (pH 6.0) containing 0.05% Tween 20 for 20 minutes. To stain for PrP, brain sections were treated with 10 µg/ml of PK for 7 minutes at RT. After 3 washes with PBS, endogenous peroxidase activity was quenched using 0.3% hydrogen peroxide in methanol for 30 minutes at RT. Non-specific binding was blocked with Protein Block Serum-Free solution (DAKO, Agilent Technologies, Santa Clara, CA, USA) for 1 hour at RT. Sections were then incubated overnight at 4 °C with the following primary antibodies, diluted in PBS containing 0.1% Tween 20: anti-PrP 1C5 antibody (1:100) (Choi et al., 2006), anti-GFAP (1:500, Merck, Darmstadt, Germany), and anti-IBA1 (1:500, EnCor Biotechnology, Gainesville, FL, USA). After rinsing with PBS, slides were incubated for 1 hour at RT with horseradish peroxidase-conjugated secondary antibodies (anti-mouse or anti-rabbit IgG) from the DAKO EnVision+ System (DAKO, Santa Clara, CA, USA). Immunoreactivity was visualized using 3,3′-diaminobenzidine (DAB; DAKO) and counterstained with ClearView Hematoxylin (BBC biochemical) and examined using a light microscope (Olympus BX51; Tokyo, Japan). For quantification, color deconvolution was performed using the H DAB vector to separate hematoxylin (blue) and DAB (brown) signals (Crowe and Yue, 2019). The deconvoluted DAB image was selected, and after setting the minimum threshold to 0, the maximum threshold was adjusted. Quantification of DAB-positive signals were performed using ImageJ Fiji. The mean percentage of DAB-positive area for PrP^Sc^, GFAP and IBA1 per given optical field were plotted as an index of their immunoreactivity (Dandi et al., 2023). For immunofluorescence staining, cells were fixed with 4% paraformaldehyde (PFA) for 10 minutes. For PrP^Sc^ detection, fixed cells were exposed to 98% formic acid for 7 minutes at RT prior to permeabilization. Cells were incubated overnight at 4 °C with mouse monoclonal anti-PrP SAF84 (1:200; Cayman Chemical Company, Ann Arbor, MI, USA) and rabbit polyclonal anti-LAMP1 (1:200, Abcam) antibodies diluted in PBST. Cells were then incubated with either Alexa Fluor 488-conjugated goat anti-rabbit IgG or Alexa Fluor 568-conjugated goat anti-mouse IgG secondary antibodies (1:1000, Invitrogen, Carlsbad, CA, USA). Nuclei were counterstained with DAPI using Vectashield antifade mounting medium (Vector Laboratories, Burlingame, CA, USA), and images were captured with an LSM700 confocal laser scanning microscope (Carl Zeiss Microscopy, Jena, Germany). For LysoTracker staining, live cells were co-incubated with LysoTracker Red DND-99 (75 nM, Thermo Fisher Scientific, Waltham, MA, USA) and Hoechst 33258 (1 µg/ml, Enzo Life Sciences) in culture medium for 30 minutes at 37 °C. Cells were then gently washed with pre-warmed PBS to remove excess dye. Images were acquired immediately using the LSM700 confocal microscope.

### Flow Cytometry

Following treatments performed as described above, cells were incubated with LysoTracker Deep Red (75 nM; Thermo Fisher Scientific) for 30 minutes at 37 °C according to the manufacturer’s instructions. Cells were then washed with DPBS, harvested, and re-suspended in FACS buffer (DPBS containing 1% BSA). Propidium iodide (PI; 1 μg/ml) was added for viability discrimination, and samples were acquired immediately. Flow cytometry analysis was performed by excluding debris based on FSC and SSC parameters, followed by singlet discrimination and gating of viable cells as PI-negative (Huang et al., 2024). LysoTracker fluorescence was quantified by histogram analysis and expressed as mean fluorescence intensity. A minimum of 10,000 live cells were analyzed per sample (*n* = 3).

### Semi-Quantitative Grading for Lesion Profile

Semi-quantitative grading of vacuolar degeneration was performed in 3 standard sites of each mouse brain using previously established methods (Kim et al., 1990). Briefly, areas of vacuolar degeneration in the mouse brains were counted in paraffin sections under light microscope (BX51, Olympus, Tokyo, Japan). Three tissue sections from each mouse were used for lesion counting. The scoring system assessed the number and size of vacuolar changes with the following scale: 0, no vacuolation; 1, a few vacuoles widely and unevenly scattered; 2, a few vacuoles evenly scattered; 3, a moderate number of vacuoles evenly scattered; and 4, marked vacuolation.

### Image Acquisition and Quantification

Images were obtained using a Zeiss LSM 700 confocal solid-state laser scanning microscope (405 nm 5 mW, 488 nm 10 mW, 555 nm 10 mW, and 639 nm 5 mW) equipped with either a Zeiss 20×/numerical aperture (NA) 1.2 or 40×/NA 1.2 water immersion objective lens, with the sequential-acquisition setting. All images were captured at a resolution of 1024 × 1024 pixels. For co-localization analysis, confocal images were processed in Fiji/ImageJ to reduce background fluorescence and isolated speckle noise using the Remove Outliers function. Colocalization was quantified within manually defined regions of interest (ROIs) corresponding to neuronal cell bodies, excluding extracellular and background regions. Manders’ coefficients were calculated using the JaCoP plugin following channel-specific threshold adjustment to minimize background contribution and identify punctate signal overlap (Han et al., 2014).

### Statistical Analysis

Data are presented as mean ± standard error mean (S.E.M). Statistical comparisons between groups were performed using 1-way analysis of variance (ANOVA) followed by Tukey’s post hoc test. All analyses were conducted using GraphPad Prism 8 program (GraphPad Software, San Diego, CA, USA) with statistical significance defined as *P* < .05.

## RESULTS

### A Single Dose of CpG ODN Prolongs Survival in 22L Scrapie-Infected Mice

**Fig. 1:**
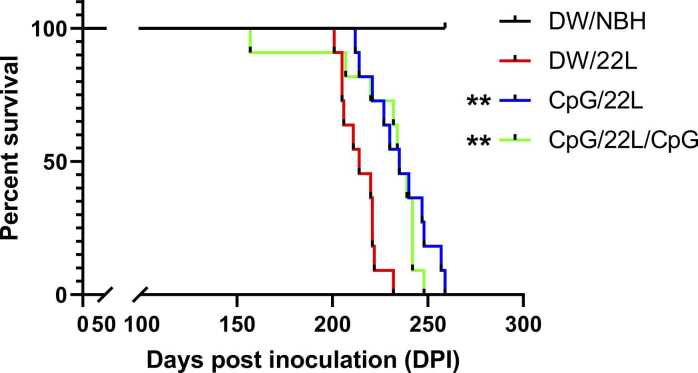
*CpG ODN treatment is associated with increased survival time in 22L scrapie-infected BALB/c mice.* Kaplan-Meier survival curves of mice assigned to 4 groups: (1) DW/NBH (uninfected control, vehicle-treated), (2) DW/22L (22L-infected, vehicle-treated), (3) CpG/22L (single CpG ODN dose 1 week prior to infection), and (4) CpG/22L/CpG (CpG ODN pretreatment plus 4 weekly booster doses post-infection). The percentage of surviving mice (y-axis) is plotted against days post-inoculation (dpi, *x*-axis). CpG-treated groups showed significantly increased survival compared to DW/22L controls, with the greatest increase observed in the CpG/22L group (*P* = .0011) and a modest but significant increase in the CpG/22L/CpG group (*P* = .0015). Statistical analysis was performed using the Kaplan-Meier method and the log-rank test (*n* = 11 per group), **P < .01. NBH, normal brain homogenate.

To evaluate the effect of CpG ODN on disease progression, we assessed the incubation period in BALB/c mice infected with the 22L scrapie strain under different treatment conditions, as detailed in the Materials and Methods section. As shown in Figure 1, the mean survival time of the 22L scrapie-infected mice (DW/22L) was 214.2 ± 2.9 dpi. Mice that received a single prophylactic dose of CpG ODN prior to infection (CpG/22L) exhibited a significantly prolonged survival time of 234.9 ± 4.9 dpi (*P* = .0011). Additional weekly CpG ODN administrations (CpG/22L/CpG) following infection did not further increase survival relative to the pretreatment group (225.5 ± 7.8 dpi), although survival was significantly increased compared to the untreated control (*P* = .0015). These results indicate that CpG ODN treatment enhances survival in scrapie-infected mice, with the most pronounced achieved through a single dose prior to infection, resulting in a 21-day increase in mean survival compared to the untreated controls.

### CpG ODN Reduces PrP^Sc^ Accumulation in the Spleen and the Brain of 22L Scrapie-Infected Mice

**Fig. 2:**
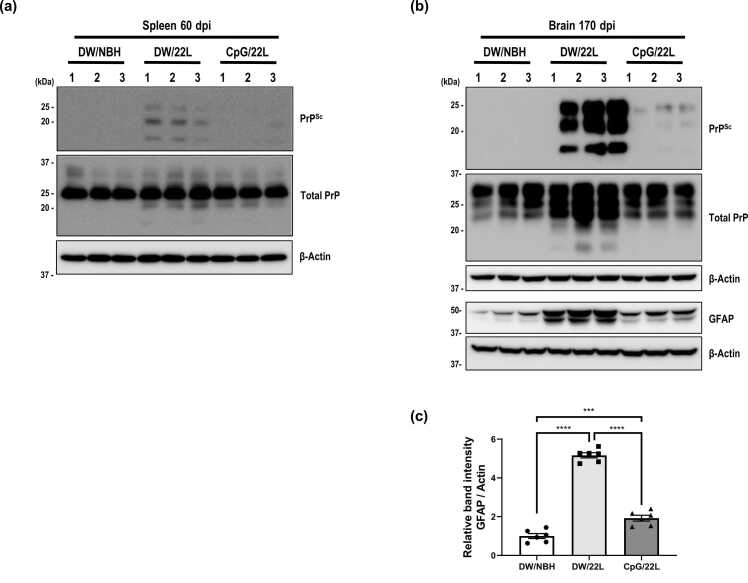
*CpG ODN reduces PrP^Sc^ accumulation and gliosis in vivo.* (a) Expression levels of PrP^Sc^ and total PrP in spleen tissues at 60 dpi, and (b) expression levels of PrP^Sc^, total PrP, and GFAP in brain tissues at 170 dpi from 22L scrapie-infected mice treated with or without CpG-ODN. Tissue homogenates were prepared from 3 groups: mice inoculated with normal brain homogenate (DW/NBH), 22L scrapie-infected mice without CpG ODN treatment (DW/22L), and 22L scrapie-infected mice with CpG ODN treatment (CpG/22L). For PrP^Sc^ detection, equal amounts of protein were incubated with proteinase K (PK, 20 μg/ml) for 1 hour. GFAP was used as a marker for gliosis in brain tissues (b). (c) Quantification of GFAP expression levels shown as a bar graph. Each group, *n* = 6. Statistical significance was determined by 1-way ANOVA with Tukey’s post hoc test. *** *P* < .001, ****P < .0001.

Next, to determine whether prolonged survival was associated with PrP^Sc^ accumulation, we performed Western blotting on spleen and brain lysates from 22L scrapie-infected mice at 60 and 170 dpi, with or without PK treatment. At 60 dpi, CpG ODN-treated mice (CpG/22L) showed reduced PrP^Sc^ levels in the spleen compared to the vehicle-treated controls (DW/22L) (Fig. 2a), suggesting that CpG ODN treatment suppresses peripheral PrP^Sc^ during early stages of infection. At this stage, PrP^Sc^ was undetectable in brain lysates from either group, likely due to the absence of neuroinvasion (data not shown). However, at 170 dpi, a subsequent reduction in PrP^Sc^ accumulation was observed in the brains of CpG/22L mice compared to DW/22L controls (Fig. 2b). Notably, the increased expression of GFAP, a hallmark of astrocytic activation commonly associated with prion-induced gliosis, was also attenuated in the brains of CpG/22L mice (Fig. 2b and c) (Tahir et al., 2022). These results suggest that CpG ODN treatment reduces PrP^Sc^ accumulation in both peripheral (spleen) and central (brain) tissues and may mitigate prion-induced gliosis, potentially contributing to the extended survival.

### CpG ODN Treatment Ameliorates Neuropathological Changes in 22L Scrapie-Infected Mice

**Fig. 3:**
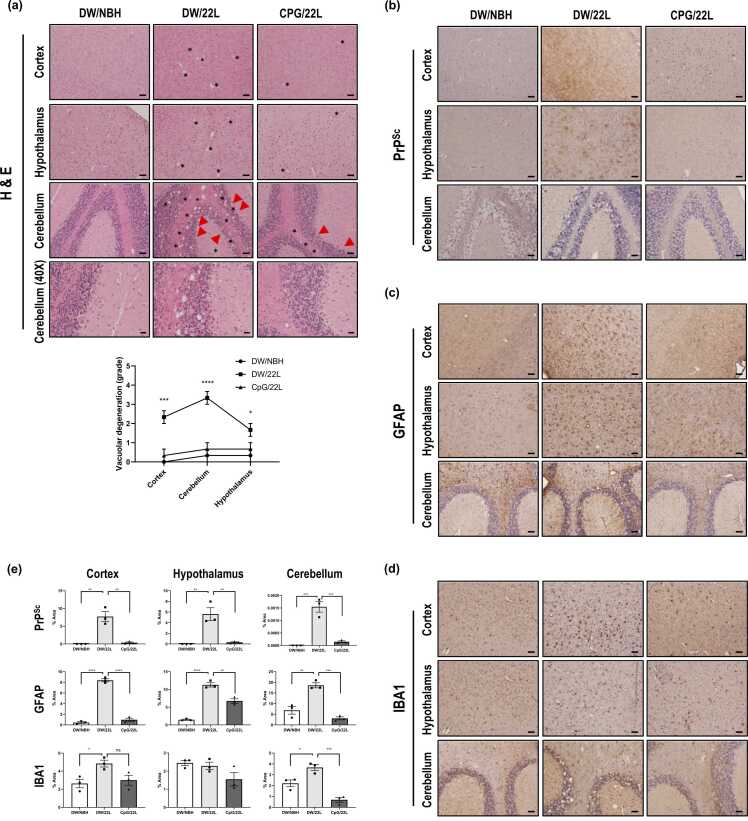
*Histopathological and quantitative analysis of prion-associated pathology in CpG ODN-treated scrapie-infected mice.* (a) Vacuolation was assessed by H&E staining. The lower panel shows lesion profiles as line graphs with symbols (mean ± S.E.M, *n* = 3). (b-d) Immunohistochemical analysis of (b) PrP^Sc^ accumulation using anti-PrP (1C5), (c) astrogliosis using anti-GFAP, and (d) microgliosis using anti-IBA1 antibodies in the cortex, hypothalamus, and cerebellum. Asterisks indicate vacuolar degeneration; arrows indicate Purkinje cell loss. (e) Quantification of immunoreactivity expressed as the mean percentage area ± S.E.M of PrP^Sc^-GFAP- or IBA1-positive staining in the indicated brain regions of scrapie-infected mice (*n* = 3 per group). All images are shown at 20X magnification (scale bar, 50 µm) or 40X magnification (scale bar, 20 µm). Statistical significance was determined by 1-way ANOVA with Tukey’s post hoc test (*P < .05, **P < .01, ***P < .001, ****P < .0001).

Vacuolation, PrP^Sc^ accumulation, and glial cell activation are well-characterized pathological hallmarks of prion diseases (Mercer and Harris, 2023). To assess these features, we performed H&E staining and immunohistochemistry using anti-PrP (3F10), anti-GFAP, and anti-IBA1 antibodies. In 22L scrapie-infected mice, extensive vacuolation was observed in both the cortex and cerebellum (asterisks), whereas CpG ODN-treated mice (CpG/22L) exhibited markedly reduced vacuolation (Fig. 3a). Notably, CpG ODN treatment preserved Purkinje cells, which were substantially diminished in untreated infected controls (arrows). Robust PrP^Sc^ deposition and marked gliosis, indicated by increased GFAP expression and IBA1 immunoreactivity, were prominent in 22L scrapie-infected mice. In contrast, CpG ODN administration significantly attenuated these pathological changes, as confirmed by quantitative image analysis (Fig. 3b-e). The effect on IBA1 immunoreactivity was region-dependent, with significant reduction observed in the cerebellum, whereas changes in the cortex and hypothalamus did not reach statistical significance (Fig. 3d and e). Together, these findings indicate that CpG ODN treatment is associated with attenuation of major neuropathological features of prion disease, which may contribute to the observed survival benefit.

### CpG ODN Treatment Enhances Autophagy Pathways in 22L Scrapie-Infected Mice

**Fig. 4:**
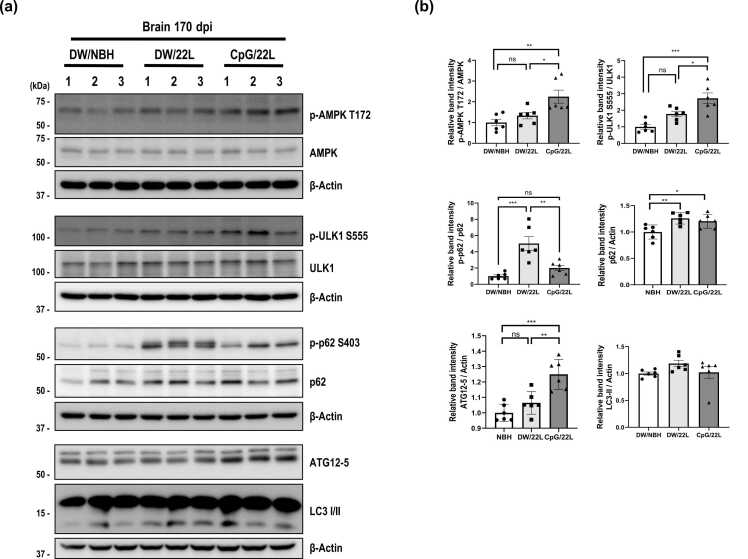
*CpG ODN induces AMPK signaling and attenuates phosphorylated p62 accumulation in 22L scrapie-infected mice.* (a) Western blot analysis of brain lysates at 170 dpi showing expression levels of p-AMPK T172, AMPK, p-ULK1 S555, ULK1, p-p62 S403, p62, ATG12–5, and LC3 I/II in brains of 22L scrapie-infected mice with or without CpG ODN at 170 dpi. (b) Relative intensity of p-AMPK, p-ULK, p62, p-p62, ATG12–5, and LC3-II represented as bar graphs (mean ± S.E.M, *n* = 6). Statistical significance was determined by 1-way ANOVA with Tukey’s post hoc test. *P < .05, **P < .01, ***P < .001. NS: not significant.

Cellular homeostasis mechanisms are essential for the clearance of misfolded proteins, with lysosome-mediated pathways—particularly autophagy—playing a central role in maintaining protein quality control (Menzies et al., 2015). In prion disease, disease progression is largely dependent on the balance between PrP^Sc^ formation and its clearance, with autophagic degradation being a key determinant. To explore the molecular mechanism underlying the prolonged survival observed in CpG/22L mice, we analyzed the expression of autophagy-related proteins in brain tissues at 170 dpi. Interestingly, Western blot analysis revealed that the CpG/22L mice exhibited significantly elevated levels of p-AMPK T172 and its downstream effector p-ULK1 S555, which initiates autophagy, compared with the DW/22L mice (Fig. 4). Furthermore, an increased ATG12-ATG5 conjugate (ATG12–5), an early component of the autophagy machinery, was observed in the brains of CpG/22L mice. Total p62 levels were modestly elevated in DW/22L mice and did not significantly decline following CpG ODN treatment. In contrast, p-p62 (S403), a modification implicated in selective autophagy–related cargo recognition (Matsumoto et al., 2011), was markedly reduced in CpG/22L mice. LC3-II levels in CpG/22L mice showed a similar downward trend but did not reach statistical significance. Together, these results indicate that CpG ODN treatment is associated with enhanced AMPK–ULK1 signaling and engagement of autophagy-related machinery in-vivo. Functional inhibitor experiments in the neuronal cell model further support the involvement of autophagy-associated degradative steps in CpG ODN–mediated PrP^Sc^ reduction.

### Autophagic Inhibition Prevents CpG ODN-Mediated PrP^Sc^ Clearance in the Scrapie-Infected Neuronal Cells

**Fig. 5:**
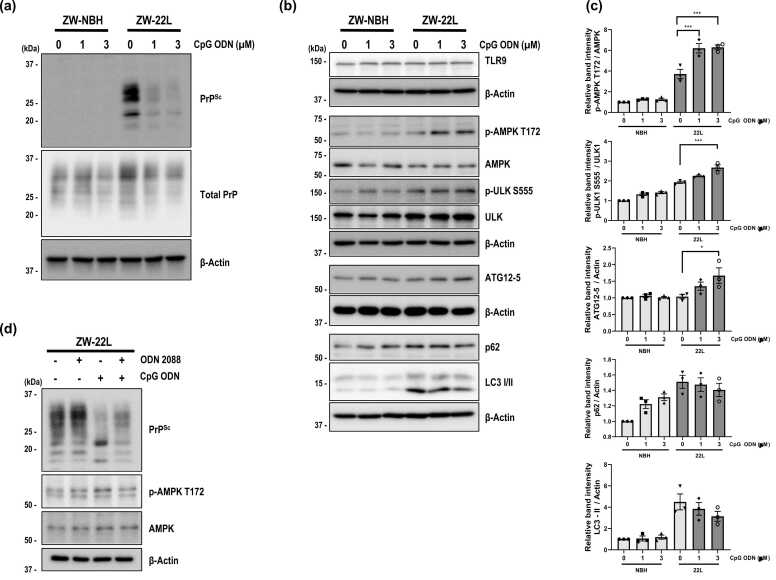
*CpG ODN reduces PrP^Sc^ accumulation and activates AMPK-associated signaling in 22L scrapie-infected neuronal cells.* (a and b) Immunoblot analysis of (a) PrP^Sc^ and total PrP, and (b) TLR9, p-AMPK T172, total AMPK, p-ULK1 S555, total ULK1, ATG12–5, total p62, and LC3 I/II in 22L scrapie-infected neuronal cells (ZW-22L) treated with CpG ODN (0, 1, or 3 µM) for 6 hours. For PrP^Sc^ detection, equal amounts of protein were incubated with proteinase K (2 µg/ml) for 1 hour. (c) Densitometric quantification of p-AMPK, p-ULK1, ATG12–5, total p62, and LC3-II levels. Data are presented as mean ± S.E.M (*n* = 3). (d) Immunoblot analysis of PrP^Sc^, p-AMPK T172, and total AMPK in ZW-22L cells treated with CpG ODN (3 µM, 6 hours) in presence or absence of the TLR9 antagonist ODN 2088 (5 µM, 7 hours). Data represents 3 independent experiments (*n* = 3). Statistical significance was determined by 1-way ANOVA with Tukey’s post hoc test (*P < .05, ***P < .001).

To validate the in vivo observations and further elucidate the mechanisms of CpG ODN-mediated PrP^Sc^ clearance, we utilized the ZW-22L neuronal cell line—a persistently 22L scrapie-infected derivative of ZW 13-2 cells that constitutively produces PK-resistant PrP^Sc^. Consistent with results obtained from scrapie-infected mouse brains, treatment of ZW-22L cells with CpG ODN resulted in a marked reduction of PrP^Sc^ levels (Fig. 5a). TLR9 protein expression was detectable in both ZW-NBH and ZW-22L neuronal cells by Western blot analysis (Fig. 5b). Previous studies have reported that neurons express TLR9 at relatively low levels compared with immune cells but remain responsive to CpG stimulation (Shintani et al., 2013, Ye et al., 2018). CpG ODN treatment was accompanied by increased p-AMPK T172 ULK1 Ser555, as well as an increase in the (ATG12–5), consistent with activation of autophagy-related signaling pathways (Fig. 5b, c). Total p62 levels did not show a significant decrease across CpG ODN concentrations (Fig. 5b, c), indicating that steady-state p62 abundance remained largely unchanged under these conditions. To assess the functional involvement of TLR9, ZW-22L cells were pretreated with the TLR9 antagonist ODN 2088 prior to CpG ODN exposure. Under these conditions, the CpG ODN–induced reduction of PrP^Sc^ was attenuated (Fig. 5d), supporting the involvement of TLR9-associated signaling in CpG ODN-mediated PrP^Sc^ reduction.

**Fig. 6:**
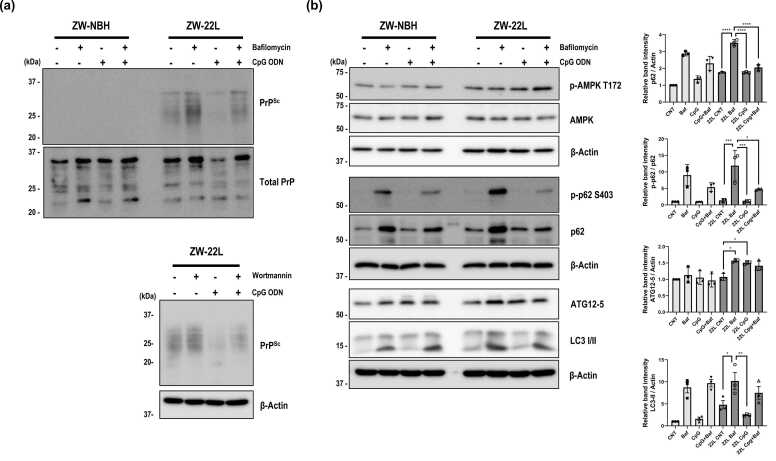
*Inhibition of autophagy abolishes CpG ODN-mediated reduction of PrP^Sc^.* Scrapie-infected (22L) or uninfected (NBH) neuronal cells were treated with CpG ODN (3 μM, 6 hours) in the presence or absence of the autophagy inhibitor bafilomycin (10 nM, 6 hours) or Wortmannin (100 nM, 6 hours). (a) Expression levels of PrP^Sc^ and total PrP were analyzed by Western blot using anti-PrP (3F10) antibody with or without proteinase K (2 μg/ml, 1 hour) digestion. Representative blots for bafilomycin (top Panel) and wortmannin (bottom panel) conditions are shown. (b) Autophagy-associated markers were analyzed by immunoblotting. Densitometric quantification is shown (mean ± S.E.M, *n* = 3). Statistical significance was determined by 1-way ANOVA with Tukey’s post hoc test (*P < .05, **P < .01, ***P < .001). Baf: Bafilomycin.

Next, to evaluate whether lysosomal degradation is required for CpG ODN-mediated PrP^Sc^ reduction, ZW-22L cells were treated with CpG ODN in the presence or absence of bafilomycin A1. As shown in Figure 6a, bafilomycin A1 blocked the CpG ODN–mediated reduction of PrP^Sc^, indicating that lysosomal degradation is required for this effect. Importantly, inhibition of early autophagy-associated steps using wortmannin likewise attenuated the CpG ODN-mediated reduction of PrP^Sc^ (Fig. 6a), supporting the requirement for an early autophagy-associated step. Autophagy-associated markers were also analyzed under lysosomal inhibition conditions (Fig. 6b). Collectively, these findings indicate that CpG ODN-mediated PrP^Sc^ reduction in scrapie-infected neuronal cells depends on both intact early autophagy-associated processes and downstream lysosomal degradation, consistent with the observed AMPK-ULK1 signaling changes in vitro and in vivo.

### CpG ODN Enhances Lysosomal Trafficking of PrP^Sc^ in Scrapie-Infected Neuronal Cells

**Fig. 7:**
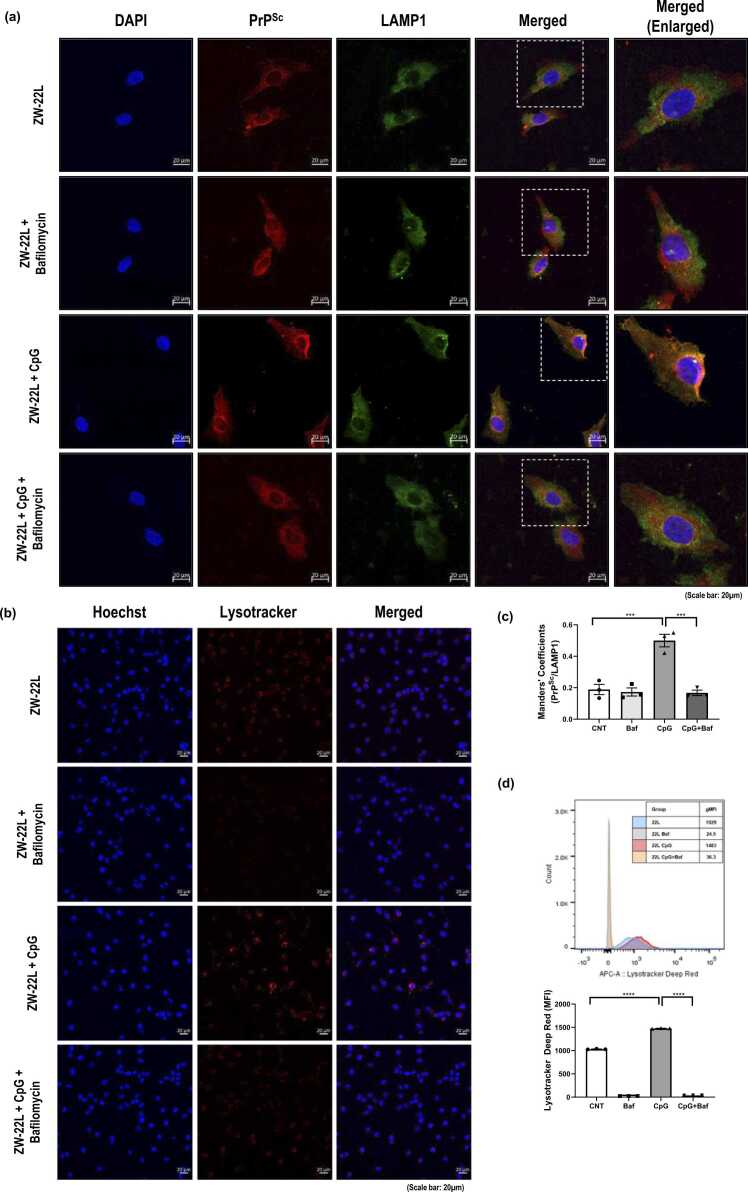
*CpG ODN enhances lysosome-associated localization of PrP^Sc^ in ZW-22L neuronal cells.* ZW-22L neuronal cells were treated with CpG ODN (1 μM, 3 hours) in the absence or presence of bafilomycin A1 (10 nM, 3 hours). (a) Confocal microscopy showing colocalization of PrP^Sc^ (red) with LAMP1 (green). Images were acquired at 40X magnification. (b) Acidic compartment signal visualized using LysoTracker Red (red), with nuclei counterstained by Hoechst (blue). Images were acquired at 20X magnification. (c) Quantification of PrP^Sc^-LAMP1 colocalization expressed as (Manders’ coefficient). (d) Flow cytometric analysis of Lysotracker Deep Red fluorescence in ZW-22L cells, quantified as mean fluorescence intensity (MFI). Data presented as mean ± S.E.M (*n* = 3). Statistical significance was determined using 1-way ANOVA with Tukey’s post hoc test (****P < .0001). Scale bar: 20 µm.

Finally, to determine whether CpG ODN treatment is associated with increased lysosomal targeting of PrP^Sc^, we examined the intracellular localization of PrP^Sc^ relative to the lysosomal marker LAMP1 by immunofluorescence staining. CpG ODN-treated ZW-22L cells exhibited increased colocalization of PrP^Sc^ with LAMP1 compared with untreated controls (Fig. 7a, b), and this increase was abolished by co-treatment with bafilomycin A1 (Fig. 7a, c). These changes were not observed in non-infected cells (Supplementary Fig. 1). In parallel, CpG ODN-treated cells showed elevated LysoTracker fluorescence intensity, indicating changes in acidic compartment signal, which was similarly reduced by bafilomycin A1 (Fig. 7b). Furthermore, flow cytometric analysis using LysoTracker Deep Red demonstrated that CpG ODN treatment significantly increased LysoTracker fluorescence intensity in ZW-22L cells, indicating increased acidic compartment signal (Fig. 7d). This increase was attenuated by bafilomycin A1, consistent with the involvement of lysosome-associated degradative processes. Together, these data, alongside the inhibitor sensitivity of PrP^Sc^ reduction (Fig. 6), are consistent with the involvement of autophagy-associated degradative processes in CpG ODN–mediated PrP^Sc^ reduction in scrapie-infected neuronal cells.

## DISCUSSION

Prion disease research has spanned several decades, yet the disease remains invariably fatal and incurable. Various therapeutic approaches have aimed to destabilize prion aggregates or prevent the conversion of cellular prion to its pathological form. However, their clinical efficacy has been limited by challenges such as poor CNS penetration, toxicity, or lack of efficacy in human trials. Modulation of intracellular clearance mechanism has recently gained interest, with chemical agents showing promising in vitro results but limited efficacy in vivo. These limitations highlight the importance to discover alternative and effective therapeutic strategies against prion diseases.

In this study, we demonstrate that a single prophylactic intraperitoneal dose of CpG ODN significantly delays disease onset, prolongs survival, and attenuates hallmark neuropathological features in a murine 22L scrapie model. These protective effects were accompanied by reduced PrP^Sc^ accumulation, less spongiform degeneration, and diminished gliosis, together indicating modulation of disease-associated pathological processes.

The observed increase in mean survival (from 214 to 235 days) is consistent with previous reports of CpG ODN-associated effects in prion disease models, which have shown variability depending on prion strain, dosing regimen, route, and timing of administration (Heikenwalder et al., 2004, Spinner et al., 2007). In our model, early administration of CpG ODN appears to intercept PrP^Sc^ propagation before substantial neuroinvasion or irreversible tissue damage occurs. Importantly, the survival benefit coincides with both symptomatic improvement and measurable reductions in PrP^Sc^ burden and gliosis.

CpG ODN treatment reduced PrP^Sc^ accumulation in both peripheral (spleen) and central (brain) compartments. In the brain, this reduction was accompanied by attenuated expression of GFAP and IBA1, markers of astrocyte and microglial activation, respectively. Since glial activation contributes significantly in prion-induced neurotoxicity (Makarava et al., 2019), these findings suggest that CpG ODNs facilitate cellular mechanisms upstream, promoting the clearance of pathological prion aggregates along with dampening of the associated neuroinflammatory response, thereby slowing disease progression. While the survival group size was modest, the observed difference remained statistically supported by log-rank analysis and aligns with prior reports of CpG ODN–associated survival extension in prion models (Sethi et al., 2002, Spinner et al., 2007). Importantly, survival outcomes were accompanied by convergent pathological and molecular changes, including reduced PrP^Sc^ burden and attenuated gliosis. Notably, a single prophylactic CpG ODN administration conferred greater benefit than repeated post-infection dosing. This pattern is biologically plausible given the timing-dependent nature of peripheral prion replication and innate immune modulation and because repeated TLR9 stimulation may engage counter-regulatory programs and/or signaling desensitization. Given the inherent variability of peripheral prion infection models, larger independent groups will be valuable in future long-term prion infection studies to further refine effect size estimates and rigorously assess reproducibility.

A key insight from this study is the association of CpG ODN treatment with AMPK signaling and engagement of autophagy-related pathways. Previous studies have suggested that TLR9 signaling can modulate metabolic and stress-responsive pathways in non-immune cells, including neurons (Shintani et al., 2013). In the present study, CpG ODN treatment increased phosphorylation of AMPK and ULK1 in scrapie-infected neuronal cells, and pharmacological inhibition of TLR9 using ODN 2088 significantly attenuated CpG ODN-mediated PrP^Sc^ reduction, supporting a functional role for TLR9 signaling in this response. Immunoblot analyses of brain tissues revealed increased p-AMPK T172, p-ULK1 S555, and ATG12–5, consistent with engagement of autophagy-related machinery. These changes were accompanied by reduced p-p62 S403, a modification associated with selective autophagy cargo handling and proteotoxic stress (Matsumoto et al., 2011). However, total p62 levels did not show a clear decrease under our experimental conditions. Because steady-state p62 abundance reflects the balance between synthesis and degradation and is influenced by stress-responsive transcriptional regulation, p62 levels may remain stable even when autophagy-associated clearance contributes to substrate handling, as emphasized in current autophagy guidelines (Klionsky et al., 2021). In scrapie-infected neuronal cells, CpG ODN-mediated reduction of PrP^Sc^ required intact degradative pathways, as inhibition of lysosomal degradation or early autophagy-associated steps using bafilomycin A1 or wortmannin, respectively, prevented PrP^Sc^ reduction. Although lysosomal blockade resulted in accumulation of p-p62 and LC3-II, these changes do not directly establish autophagic flux and may reflect cargo load or pathway perturbations. Therefore, conclusions regarding autophagy involvement in the present study are based primarily on functional PrP^Sc^ outcomes and their sensitivity to pharmacological perturbation of early and late autophagy-associated steps. Consistent with this interpretation, CpG ODN–treated neuronal cells exhibited increased association of PrP^Sc^ with lysosomal markers, suggesting enhanced lysosomal engagement. While these findings support a role for TLR9 signaling, additional TLR9-independent or parallel signaling mechanisms may also contribute to CpG ODN–mediated effects, particularly in the context of systemic administration and neuronal stress responses.

Autophagy impairment is a hallmark of prion pathogenesis, contributing to the accumulation of PrP^Sc^ along with autophagy adapter proteins such as p62/SQSTM1 (Homma et al., 2014, Thellung et al., 2018). In this context, our findings indicate that CpG ODN–mediated PrP^Sc^ reduction depends on intact autophagy-associated processes and lysosomal degradation and is associated with activation of TLR9-linked AMPK–ULK1 signaling. While pharmacological inhibition cannot fully exclude the contribution from additional pathways, the observed sensitivity of PrP^Sc^ reduction to perturbation of autophagy-associated steps provides functional support for the involvement of autophagy-associated mechanisms. Accordingly, our data support the involvement of autophagy-associated degradative processes in PrP^Sc^ clearance but do not establish enhanced autophagic flux per se. Future studies incorporating direct autophagic flux assays will be required to determine whether CpG ODN modulates autophagic flux in addition to engaging autophagy-associated machinery.

Therapeutic strategies for prion disease have traditionally focused on inhibiting the conversion of PrP^C^ to PrP^Sc^ or facilitating the clearance of pathogenic aggregates. More recently, induction of autophagy has emerged as a promising approach for degrading intracellular prion aggregates. However, the in vivo efficacy of autophagy inducers has been variable. Rapamycin, an mTOR inhibitor, promotes autophagic flux, reduces prion burden, and modestly extends survival in some prion models (Sarkar et al., 2009, Thellung et al., 2018). whereas mTOR-independent compounds like trehalose demonstrate PrP^Sc^ clearance in vitro but fail to produce survival benefits in vivo (Aguib et al., 2009). Unlike rapamycin and radotinib (Choi et al., 2023), CpG ODN treatment was associated with engagement of autophagy-related pathways through an mTOR-independent, AMPK-driven mechanism.

In this study, CpG ODN was employed to examine whether engagement of neuronal TLR9 can modulate intracellular clearance pathways in prion-infected neuronal cells. Accordingly, the observed reduction in PrP^Sc^ following CpG ODN treatment is therefore consistent with regulation of cellular proteostasis mechanisms rather than PrP^Sc^-specific targeting. Autophagy and related protein quality control pathways are broadly implicated in the pathogenesis of multiple neurodegenerative proteinopathies. In this context, TLR9 agonist CpG ODN has been reported to ameliorate amyloid-related pathology in Alzheimer’s disease models, including in non-human primates, and to enhance microglial clearance of oligomeric amyloid-β (Patel et al., 2021, Scholtzova et al., 2009). These observations suggest that CpG–TLR9 signaling may influence aggregate-associated pathology beyond prion disease. Nevertheless, whether CpG ODN exerts comparable effects on other pathogenic aggregates, such as amyloid-β or α-synuclein, within neuronal or mixed neural cell systems requires direct evaluation in disease-relevant models and warrants further investigation.

CpG ODNs have been investigated in contexts such as cancer immunotherapy and infectious disease vaccines; yet, their application to prion diseases poses challenges due to long, clinically silent incubation periods and typically late-stage diagnosis. Given the extended disease course, sustained or repeated CpG ODN administration may be required to maintain biological activity, raising practical considerations for long-term safety and tolerability. Importantly, the prophylactic design of this study limits direct clinical translatability, as prion diseases are typically diagnosed after symptom onset. Accordingly, the present work should be regarded as a pathway-focused proof-of-concept demonstrating the capacity of CpG ODN to modulate disease-relevant pathways, providing a foundation for future studies employing post-symptomatic or therapeutic treatment paradigms to more directly evaluate clinical applicability.

A major challenge to the clinical application of CpG ODN in prion disease is its limited penetration into the CNS (Ribes et al., 2014), which may require advanced delivery strategies. Nanoparticle-based systems, including liposomes, micelles, dendrimers, and solid-lipid nanoparticles, have shown potential to enhance delivery across the blood–brain barrier in neurodegenerative disease models (Haro-Martínez et al., 2025). Despite this limitation, the present study detected significant molecular alterations in the brain, including increased activity of AMPK and ULK1 and decreased phosphorylation of p62, alongside corresponding effects in prion-infected neuronal cells. These results suggest that CpG ODN treatment may influence CNS pathways indirectly, rather than through direct brain penetration. Although CpG ODN does not readily cross the blood-brain barrier, peripheral TLR9 activation can modulate CNS function via immune-to-brain signaling mechanisms, such as altered immune cell trafficking, modulation of microglial activation states, and perivascular interactions. Such peripheral-to-central communication has been shown to modify CNS inflammatory and phagocytic responses in disease models without direct CpG ODN entry into the brain (Ribes et al., 2009, Ribes et al., 2014). Taken together, the observed changes in autophagy-associated signaling may reflect downstream consequences of peripheral immune modulation.

Overall, the present findings indicate that CpG ODN treatment is associated with modulation of autophagy-related pathways, including AMPK-ULK1 signaling, and requires intact autophagy-associated and lysosomal processes for PrP^Sc^ reduction. These effects were accompanied by reduced glial activation and prolonged survival in a prion disease model. While further work is required to establish pathway specificity and therapeutic efficacy, the data underscore the potential relevance of autophagy-associated mechanisms in prion pathogenesis and support continued investigation of CpG ODN–based modulation of intracellular clearance pathways. Future translational studies should focus on delivery optimization, dosing strategies, and CNS bioavailability to fully realize the therapeutic potential of CpG ODNs.

## Funding and Support

This work was supported by the National Research Foundation of Korea (NRF) grant funded by the Korea government (MSIT) (RS-2023-00247903).

## CRediT authorship contribution statement

**Mohd Najib Mostafa:** Writing – review & editing, Writing – original draft, Validation, Methodology. **Byungki Jang:** Writing – review & editing, Methodology. **Mo-Jong Kim:** Methodology. **Myung-Ju Choi:** Validation, Methodology. **Younghee Lee:** Writing – review & editing. **Yong-Sun Kim:** Writing – review & editing, Conceptualization. **Hyung-Joo Kwon:** Writing – review & editing, Funding acquisition, Conceptualization. **Eun-Kyoung Choi:** Supervision, Writing – review & editing, Project administration, Conceptualization.

## Declaration of Competing Interests

The authors declare that they have no known competing financial interests or personal relationships that could have appeared to influence the work reported in this paper.

## Data Availability

Data for this study are available from the corresponding author on reasonable request.

## Appendix A Supplementary material

Supplementary Figure 1. **Lack of PrP**^**Sc**^**and LAMP1 colocalization in non-infected ZW cells**. Non-infected ZW-NBH cells were treated with CpG ODN (1 μM, 3 h) in the absence or presence of bafilomycin A1 (10 nM, 3 h). Confocal microscopy was performed to visualize PrP^Sc^ (red) and LAMP1 (green). Images confirm no detectable colocalization of PrP and LAMP1 in the absence of prion infection. Images were acquired at 40X magnification (scale bar: 20 µm)

## Footnotes

Appendix A Supplemental material associated with this article can be found online at: doi:10.1016/j.mocell.2026.100335.
