# Supplementary material for: CpG oligodeoxynucleotide reduces PrPSc accumulation and prolongs survival in prion-infected mice
Source: Mol Cells. 2026 Mar 3;49(5):100335. doi: 10.1016/j.mocell.2026.100335 (PMC13052180; doi:10.1016/j.mocell.2026.100335)
Supplement: Supplementary file 1 — Supplementary Figure 1. Lack of PrPScand LAMP1 colocalization in non-infected ZW cells. Non-infected ZW-NBH cells were treated with CpG ODN (1 μM, 3 h) in the absence or presence of bafilomycin A1 (10 nM, 3 h). Confocal microscopy was performed to visualize PrPSc (red) and LAMP1 (green). Images confirm no detectable colocalization of PrP and LAMP1 in the absence of prion infection. Images were acquired at 40X magnification (scale bar: 20 µm) [file mmc1.pptx]

## Slide 1
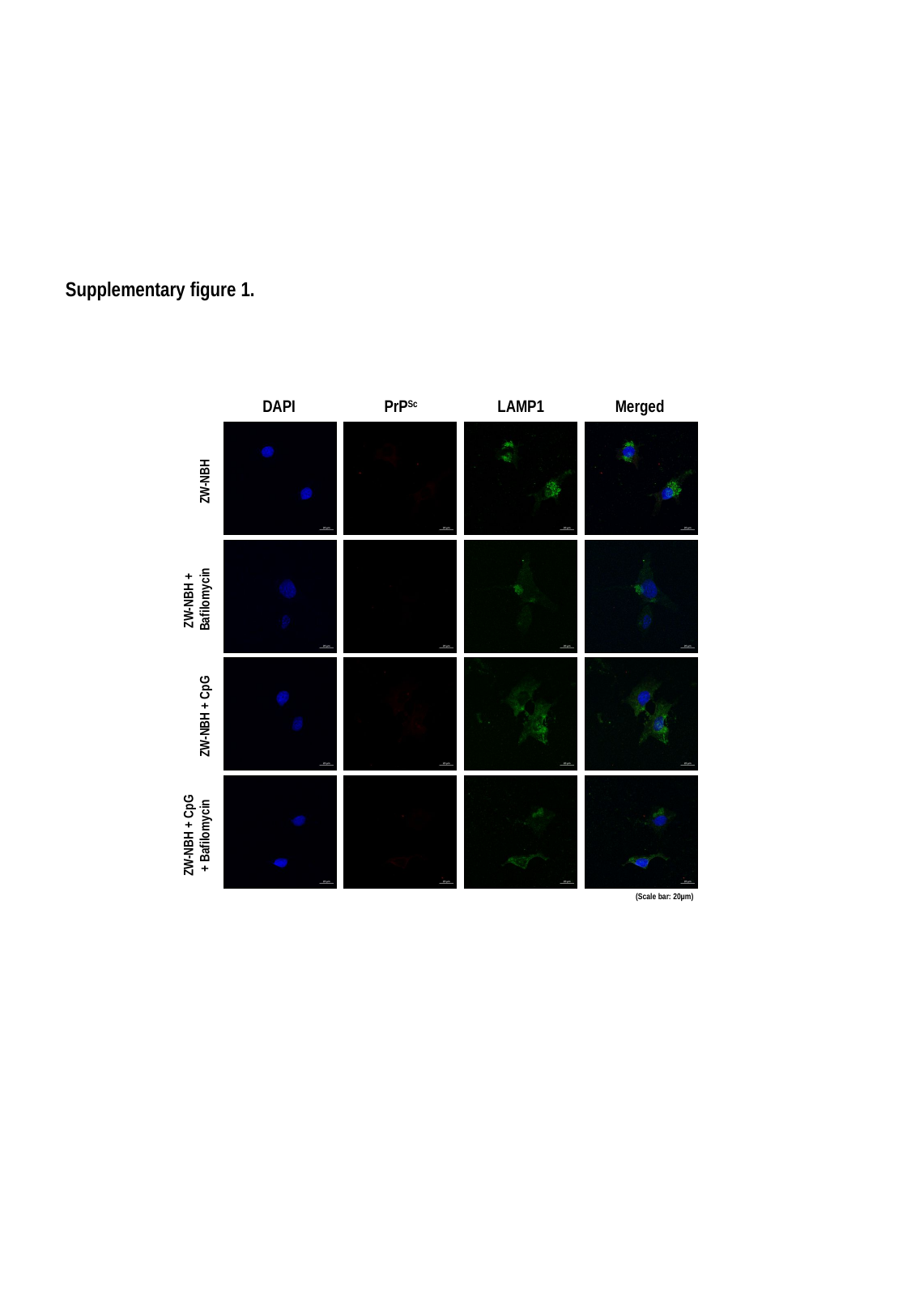

Supplementary figure 1.
PrPSc
DAPI
LAMP1
Merged
ZW-NBH
ZW-NBH +
Bafilomycin
ZW-NBH + CpG
ZW-NBH + CpG + Bafilomycin
(Scale bar: 20µm)
