# Supplementary material for: CpG oligodeoxynucleotide reduces PrPSc accumulation and prolongs survival in prion-infected mice
Source: Mol Cells. 2026 Mar 3;49(5):100335. doi: 10.1016/j.mocell.2026.100335 (PMC13052180; doi:10.1016/j.mocell.2026.100335)
Supplement: Supplementary file 2 — Supplementary material [file mmc2.pptx]

## Slide 1
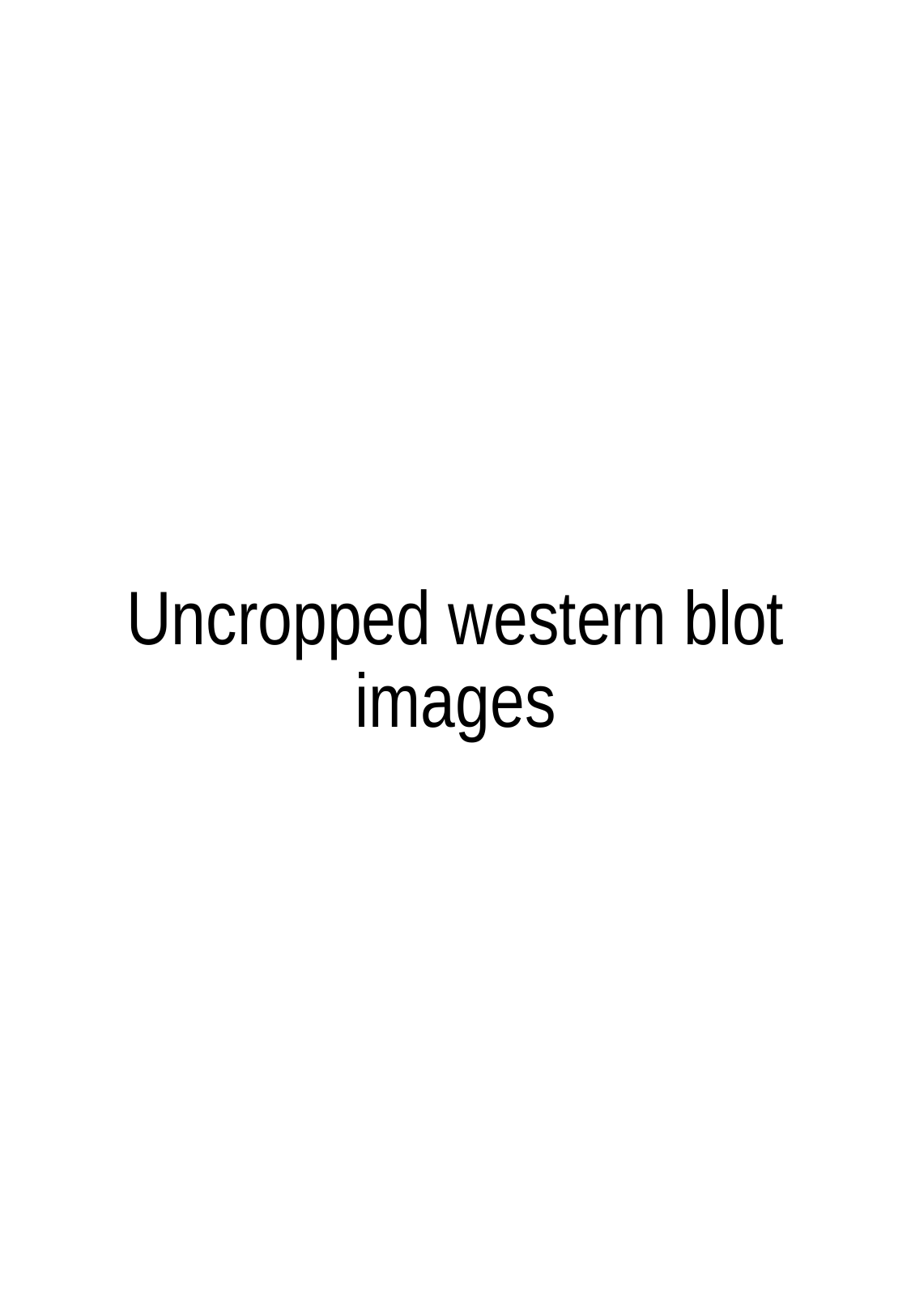

# Uncropped western blot images

## Slide 2
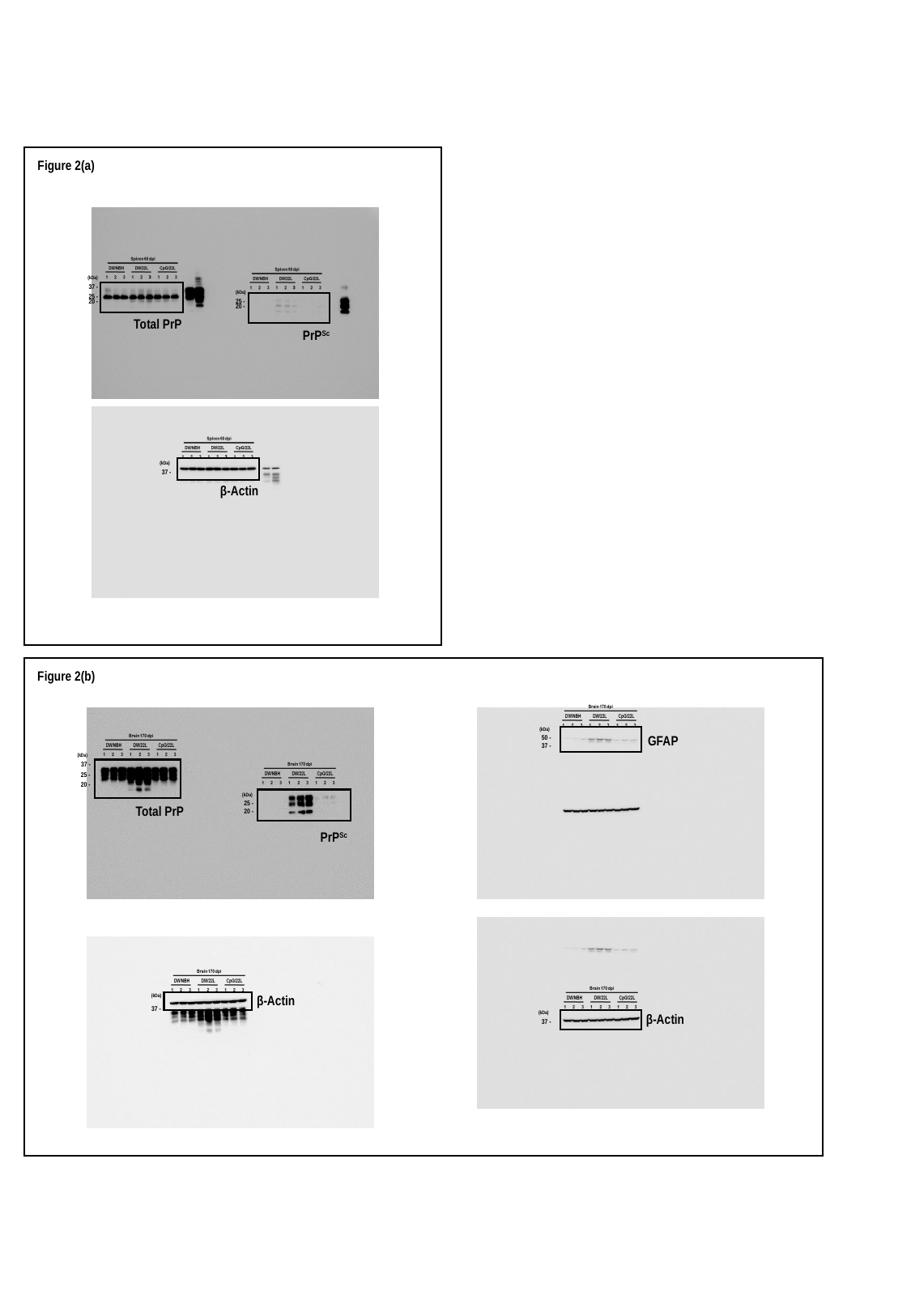

Figure 2(a)
(kDa)
37 -
(kDa)
25 -
25 -
20 -
20 -
Total PrP
PrPSc
(kDa)
37 -
β-Actin
Figure 2(b)
(kDa)
GFAP
50 -
37 -
(kDa)
37 -
25 -
20 -
(kDa)
25 -
Total PrP
20 -
PrPSc
β-Actin
(kDa)
37 -
(kDa)
β-Actin
37 -

## Slide 3
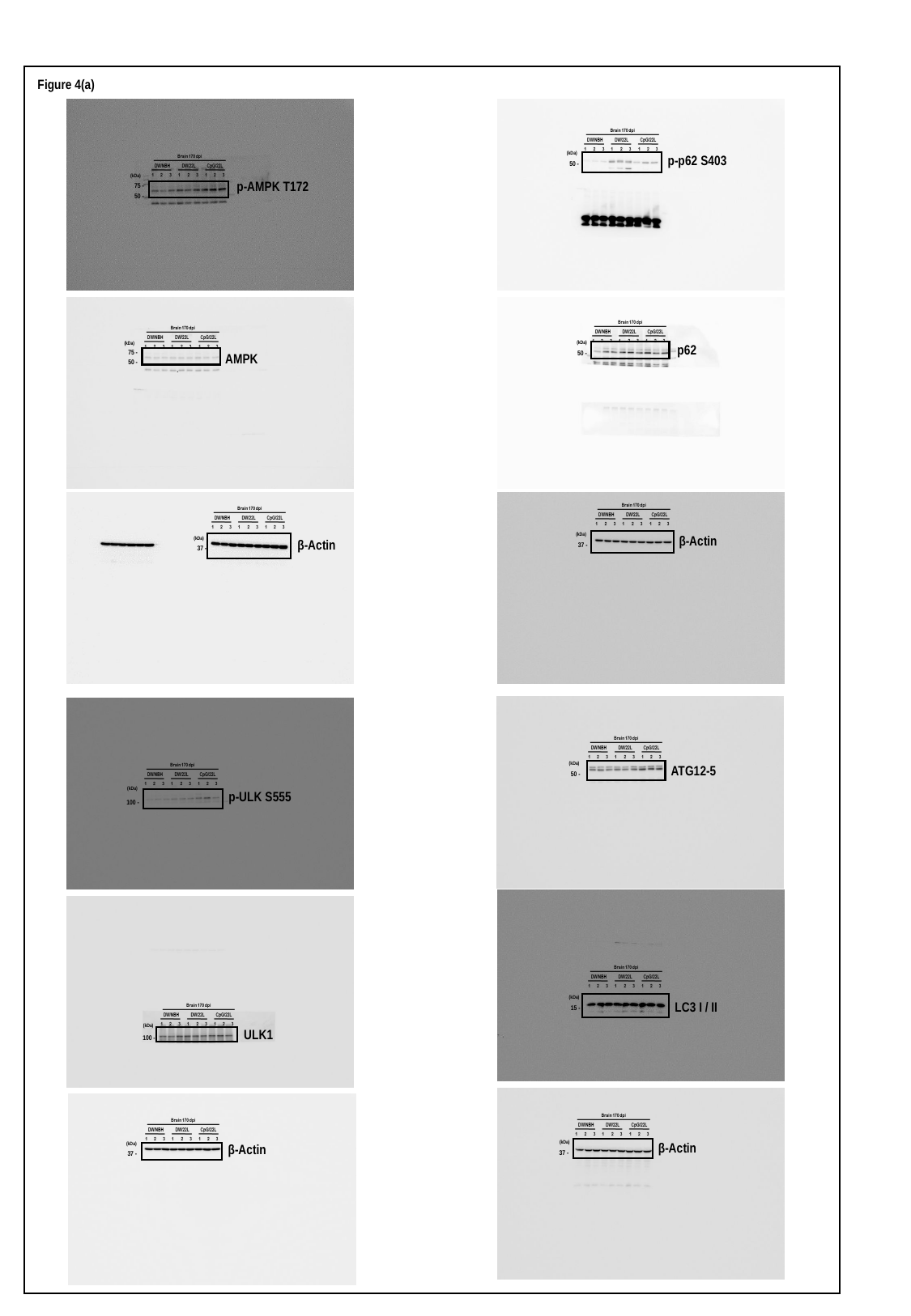

Figure 4(a)
(kDa)
p-p62 S403
50 -
(kDa)
p-AMPK T172
75 -
50 -
(kDa)
(kDa)
p62
75 -
50 -
AMPK
50 -
(kDa)
β-Actin
(kDa)
β-Actin
37 -
37 -
(kDa)
ATG12-5
50 -
(kDa)
p-ULK S555
100 -
(kDa)
LC3 I / II
15 -
(kDa)
ULK1
100 -
(kDa)
β-Actin
β-Actin
(kDa)
37 -
37 -

## Slide 4
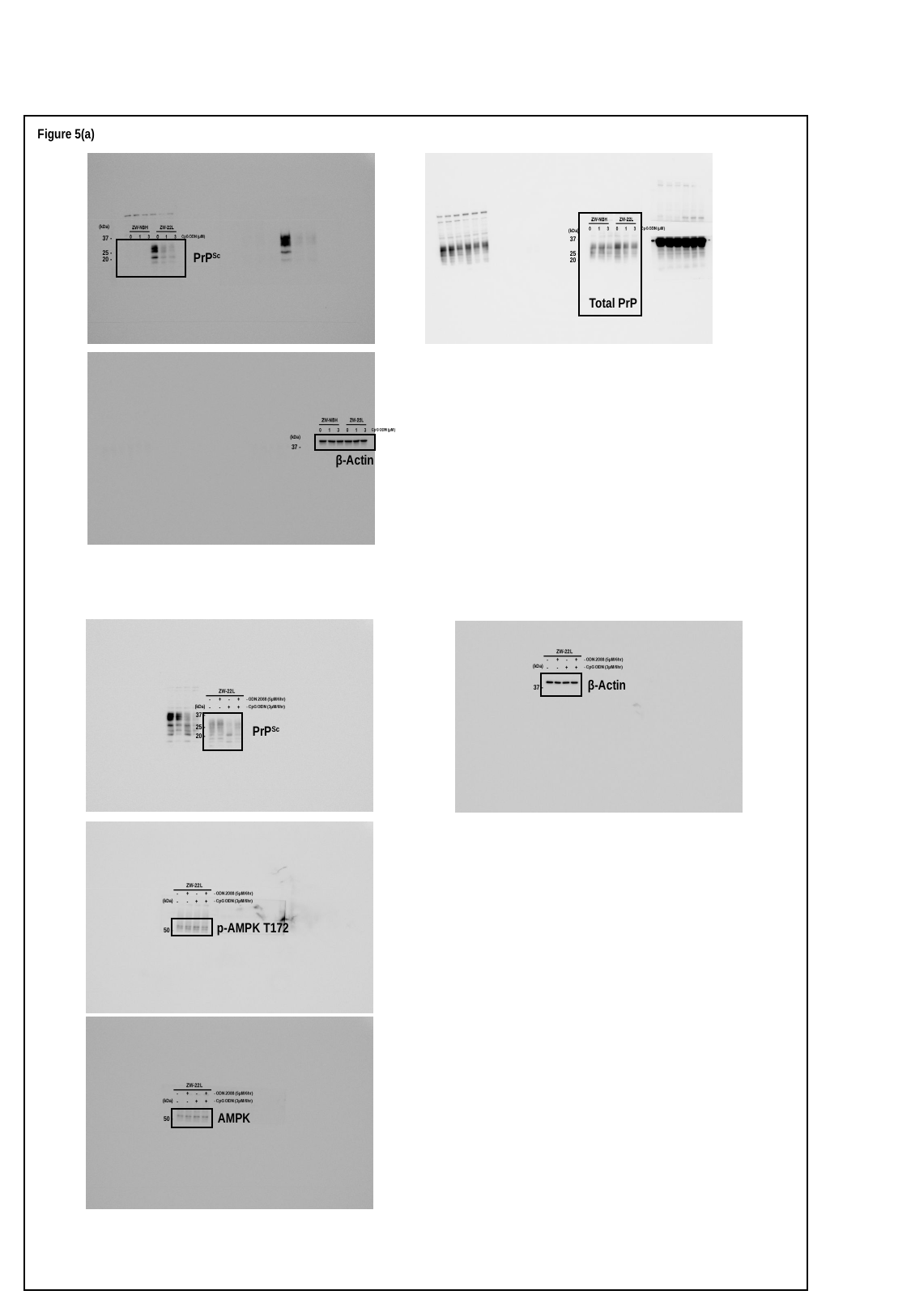

Figure 5(a)
(kDa)
(kDa)
37 -
37 -
25 -
PrPSc
25 -
20 -
20 -
Total PrP
(kDa)
37 -
β-Actin
(kDa)
β-Actin
37 -
(kDa)
37 -
PrPSc
25 -
20 -
(kDa)
p-AMPK T172
50 -
(kDa)
AMPK
50 -

## Slide 5
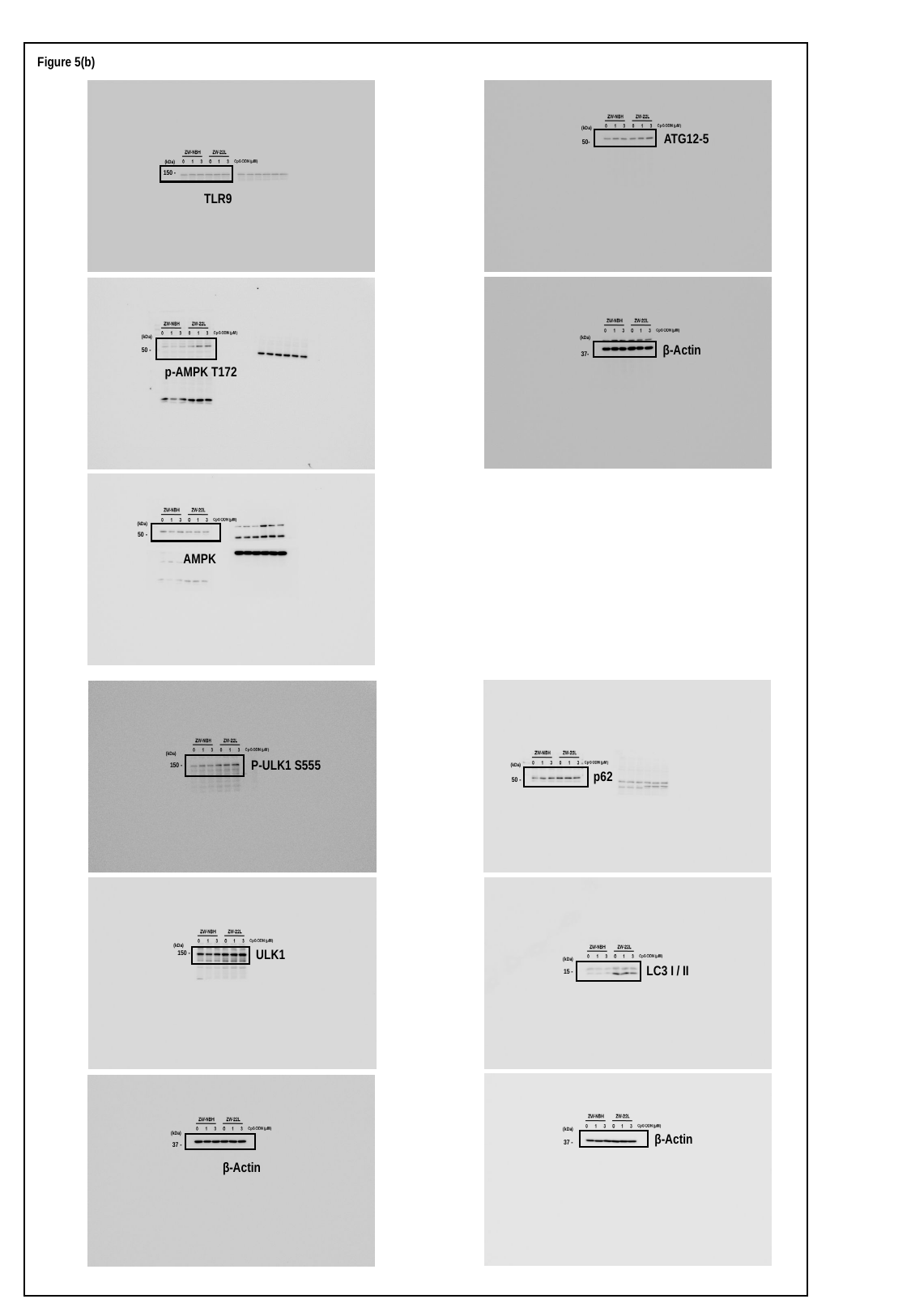

Figure 5(b)
(kDa)
ATG12-5
50-
(kDa)
150 -
TLR9
(kDa)
(kDa)
β-Actin
50 -
37-
p-AMPK T172
(kDa)
50 -
AMPK
(kDa)
P-ULK1 S555
150 -
(kDa)
p62
50 -
(kDa)
ULK1
150 -
(kDa)
LC3 I / II
15 -
(kDa)
(kDa)
β-Actin
37 -
37 -
β-Actin

## Slide 6
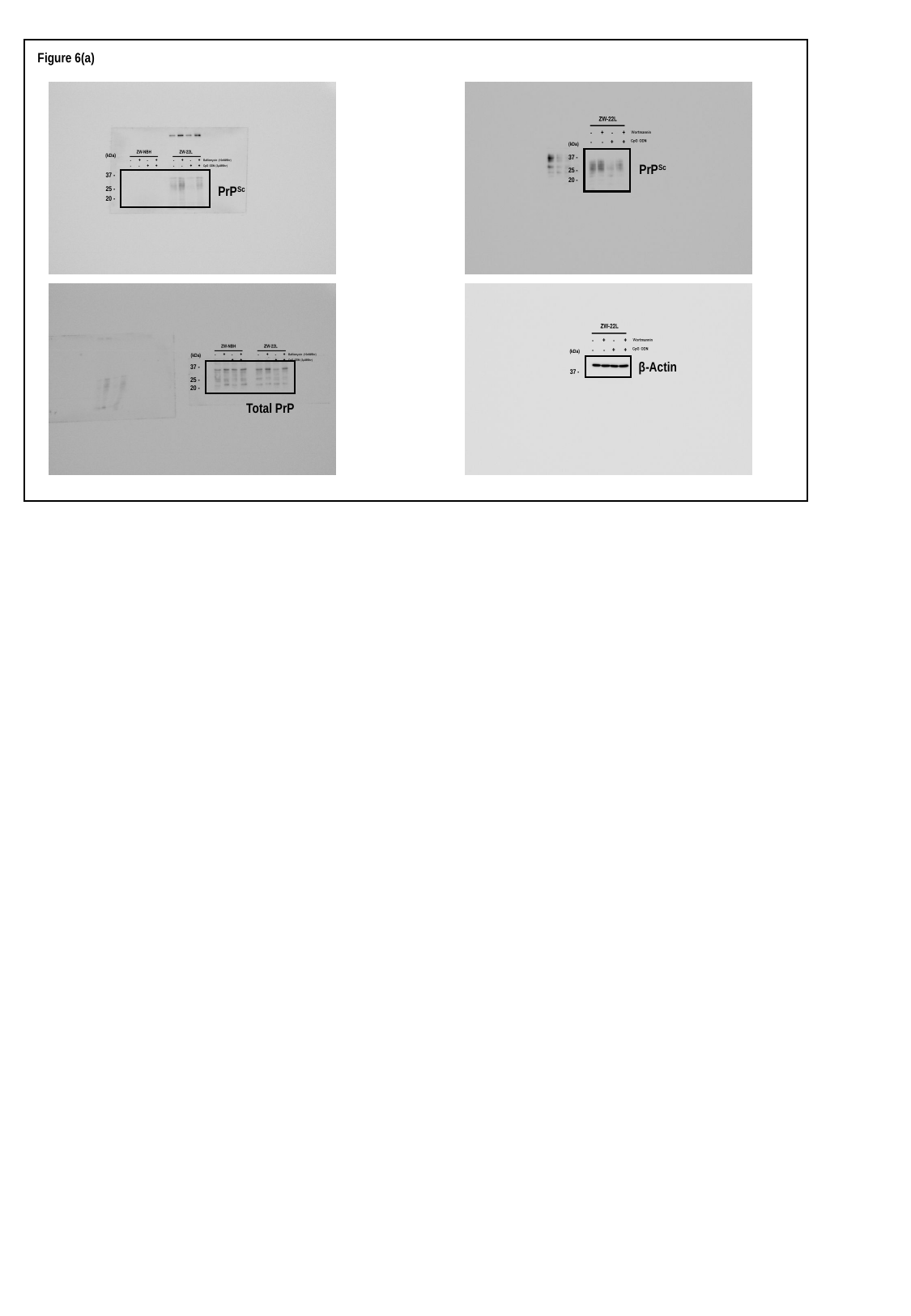

Figure 6(a)
(kDa)
(kDa)
37 -
PrPSc
25 -
37 -
20 -
PrPSc
25 -
20 -
(kDa)
(kDa)
β-Actin
37 -
37 -
25 -
20 -
Total PrP

## Slide 7
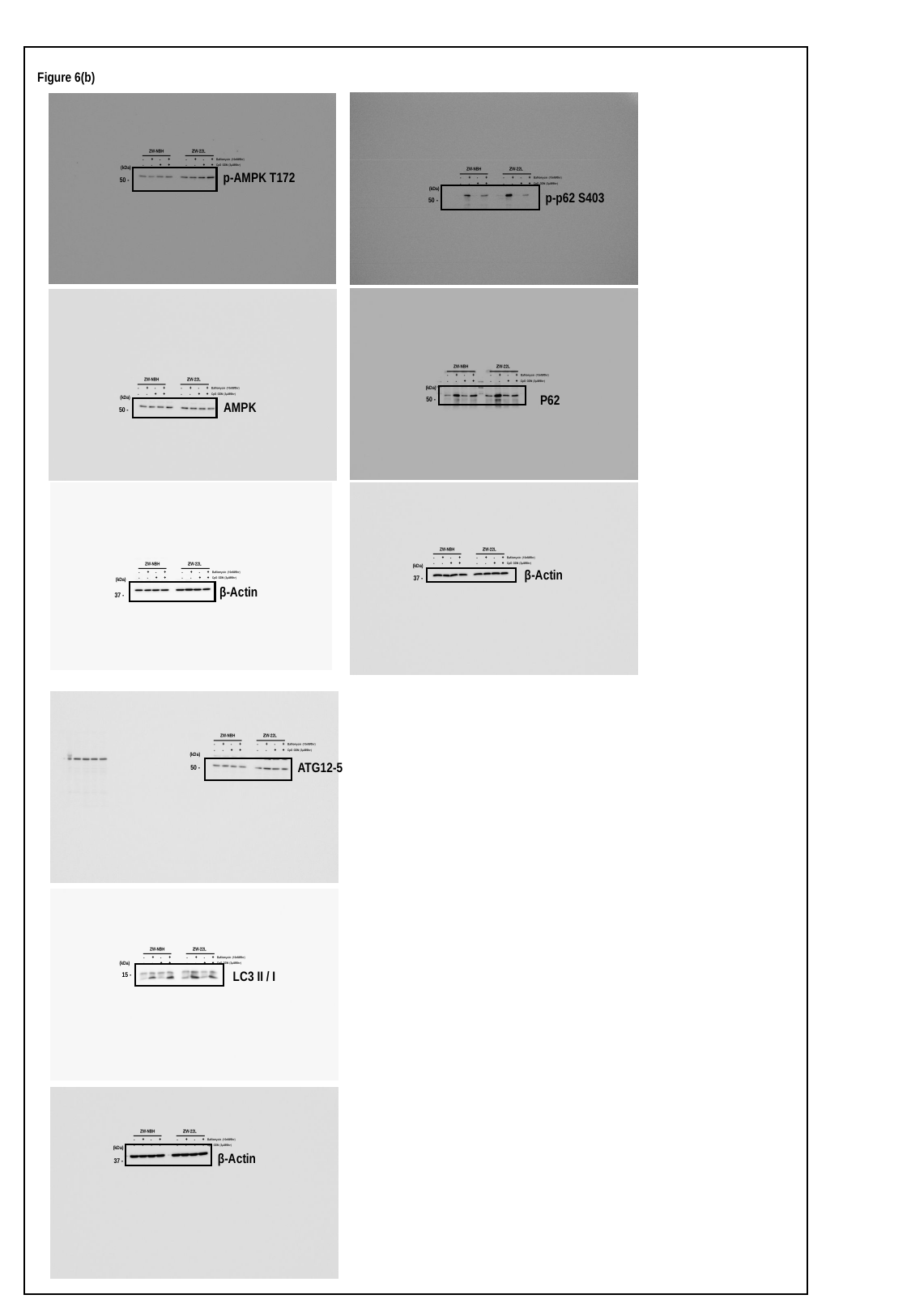

Figure 6(b)
(kDa)
p-AMPK T172
50 -
(kDa)
p-p62 S403
50 -
(kDa)
P62
(kDa)
50 -
AMPK
50 -
(kDa)
β-Actin
37 -
(kDa)
β-Actin
37 -
(kDa)
ATG12-5
50 -
(kDa)
LC3 II / I
15 -
(kDa)
β-Actin
37 -
